# Caribou, water, and ice – fine-scale movements of a migratory arctic ungulate in the context of climate change

**DOI:** 10.1186/s40462-016-0079-4

**Published:** 2016-04-20

**Authors:** Mathieu Leblond, Martin-Hugues St-Laurent, Steeve D. Côté

**Affiliations:** Caribou Ungava, Département de biologie, and Center for Northern Studies, Université Laval, 1045 avenue de la Médecine, Québec, QC G1V 0A6 Canada; Caribou Ungava, Département de biologie, chimie et géographie, Center for Northern Studies, and Center for Forest Research, Université du Québec à Rimouski, 300 allée des Ursulines, Rimouski, QC G5L 3A1 Canada

**Keywords:** Behaviour, Climate, Global change, Long-distance migration, Migratory caribou, Movements, Phenology, *Rangifer tarandus*, Scales, Space use, Step selection function

## Abstract

**Background:**

Freshwater lakes and rivers of the Northern Hemisphere have been freezing increasingly later and thawing increasingly earlier during the last century. With reduced temporal periods during which ice conditions are favourable for locomotion, freshwater bodies could become impediments to the inter-patch movements, dispersion, or migration of terrestrial animals that use ice-covered lakes and rivers to move across their range. Studying the fine-scale responses of individuals to broad-scale changes in ice availability and phenology would help to understand how animals react to ongoing climate change, and contribute to the conservation and management of endangered species living in northern environments. Between 2007 and 2014, we equipped 96 migratory caribou *Rangifer tarandus caribou* from the Rivière-aux-Feuilles herd in northern Québec (Canada) with GPS telemetry collars and studied their space use. We measured contemporary (digital MODIS maps updated every 8 days, 2000–2014) and historical (annual observations, 1947–1985) variations in freshwater-ice availability and evaluated the concurrent responses of caribou to these changes.

**Results:**

Ice had a positive influence on caribou movement rates and directionality, and caribou selected ice and avoided water when moving across or in the vicinity of large water bodies. When ice was unavailable, caribou rarely swam across (6 % of crossings) and frequently circumvented water bodies for several km. Although ice phenology did not change significantly during our study, climate projections indicated that ice availability could decrease considerably before the end of the century, generating a ~28 % increase in distance travelled by caribou during the early spring and fall migrations.

**Conclusions:**

We demonstrated that ice availability influenced the movements of a migratory arctic ungulate. Warmer air temperatures in the Arctic will undoubtedly modify the phenology of ice forming on freshwater lakes and rivers. If migratory caribou are unable to adjust the timing of their migrations, they could be forced to circumvent unfrozen water bodies more frequently and over broader areas, which may increase the distance, time, and energy they use to reach wintering areas. The long-term conservation of wide-ranging species will ultimately depend on our ability to identify the fine-scale behavioural reactions of individuals to broad-scale changes in climate and land use.

**Electronic supplementary material:**

The online version of this article (doi:10.1186/s40462-016-0079-4) contains supplementary material, which is available to authorized users.

## Background

Climate is changing rapidly [1], and the regional distribution of many plant and animal taxa has begun to shift spatially in response to the changing environment [2, 3]. Much research has focused in recent years on the ecological impacts of climate change on species or communities [4, 5], and although changes in the distribution of species are likely the mirror of individual responses to climate change [6], studies linking fine-scale individual behaviour with broad-scale climatic trends are slow to follow (but see, e.g., [7–9]). Studying individual responses to climate may help understand how species will react, and potentially adapt to climate change; this knowledge would be of paramount scientific, conservation, and management value [10].

High-latitude environments are disproportionately affected by climate change [11, 12]. Arctic and subarctic species now cope with warmer temperatures, altered precipitation patterns, and more frequent extreme weather events [1]. Numerous studies have demonstrated that Arctic perennial sea ice is declining rapidly [13, 14], affecting the ecology and demography of pagophilic (i.e., ice-dependent) species such as the polar bear (*Ursus maritimus* [15]), Pacific walrus (*Odobenus rosmarus divergens* [16]), and hooded seal (*Cystophora cristata* [17]). Accumulating evidence also suggests that the phenology of freshwater-ice formation and ablation in northern regions is changing (see [18, 19] and references therein). For example, Magnuson et al. [18] analyzed time series of ice extents on water bodies of the Northern Hemisphere and found that lakes and rivers had increasingly earlier breakup and later freezing dates during the 1846–1995 period. These seasonal processes may influence non-pagophilic terrestrial species that use ice on inland water bodies to move across their range or colonize new areas [20, 21] by impeding their movements [22], altering the timing of their migrations [23], or increasing their risks of drowning [21].

We studied the responses of migratory caribou (*Rangifer tarandus caribou*) to trends in the availability of ice on lakes and rivers in an Arctic/subarctic region to assess potential mechanisms linking fine-scale individual behaviour to broad-scale climatic conditions. Predicting changes in caribou migration routes in response to climate change would aid conservation efforts for the species (notably regarding planning of human developments [24]) as well as ensure long-term sustainability of the Aboriginal hunt [25]. Caribou perform one of the most impressive terrestrial long-distance migrations in the world [26]. In Northern Québec (Canada), caribou travel distances up to 1 000 km from their calving grounds in the Ungava Peninsula to their wintering areas in the boreal forest. Along the way, they encounter many lakes and rivers, which are highly abundant in the region [27]. The potential consequences of crossing water bodies for caribou could depend on the availability of ice. Caribou are often observed swimming across open water bodies [28], but this type of locomotion is much less efficient and more energetically costly than walking [29]. Frozen water bodies are plane, wind-swept surfaces with packed snow and increased visibility that make them ideal substrates to move rapidly during travel. On the other hand, frozen lakes are entirely devoid of vegetation, and represent large open areas that may facilitate detection by predators [30] and hunters [31]. Thus, caribou encountering large water bodies may have to trade off energy maintenance (i.e., by crossing) with foraging opportunities (i.e., by going around), as well as mortality risk (including risks of drowning [21]).

Our objectives were four-fold. First, we analyzed contemporary (2000–2014) and historical (1947–1985) thawing and freezing trends of lakes and rivers in the range of the Rivière-aux-Feuilles (RAF) caribou herd in Northern Québec (Canada). We predicted that these local trends would reflect results obtained by Magnuson et al. [18] at much larger spatiotemporal scales. Second, we assessed the responses of caribou to ice and water availability by studying their space use and movements on and around lakes and rivers during 8 years (2007–2014). This was done at a fine spatiotemporal scale using GPS-collared animals and regularly updated (i.e., every 8 days) 500-m resolution ice maps. We predicted that caribou would select ice during their movements to travel more rapidly and directly, and to reduce the energetic costs associated with swimming [29]. We also predicted that caribou would circumvent water bodies more frequently when ice was unavailable [32]. Third, we assessed the relationship between fine-scale, intra-annual variability in caribou behaviour and broad-scale, inter-annual changes in ice phenology. We predicted that caribou would adjust their behaviour (e.g., avoid water more strongly) according to trends in breakup and freeze dates observed during the study. Finally, we projected the contemporary movements of caribou within a gradient of future ice phenology scenarios to determine the potential consequences of climate change on the movements of caribou during the next 25 – 50 years. We predicted that future climatic conditions could force caribou to circumvent unfrozen water bodies more often, resulting in an increase in the total distance travelled by caribou to reach wintering and calving areas.

## Methods

### Study area and caribou herd

**Fig. 1:**
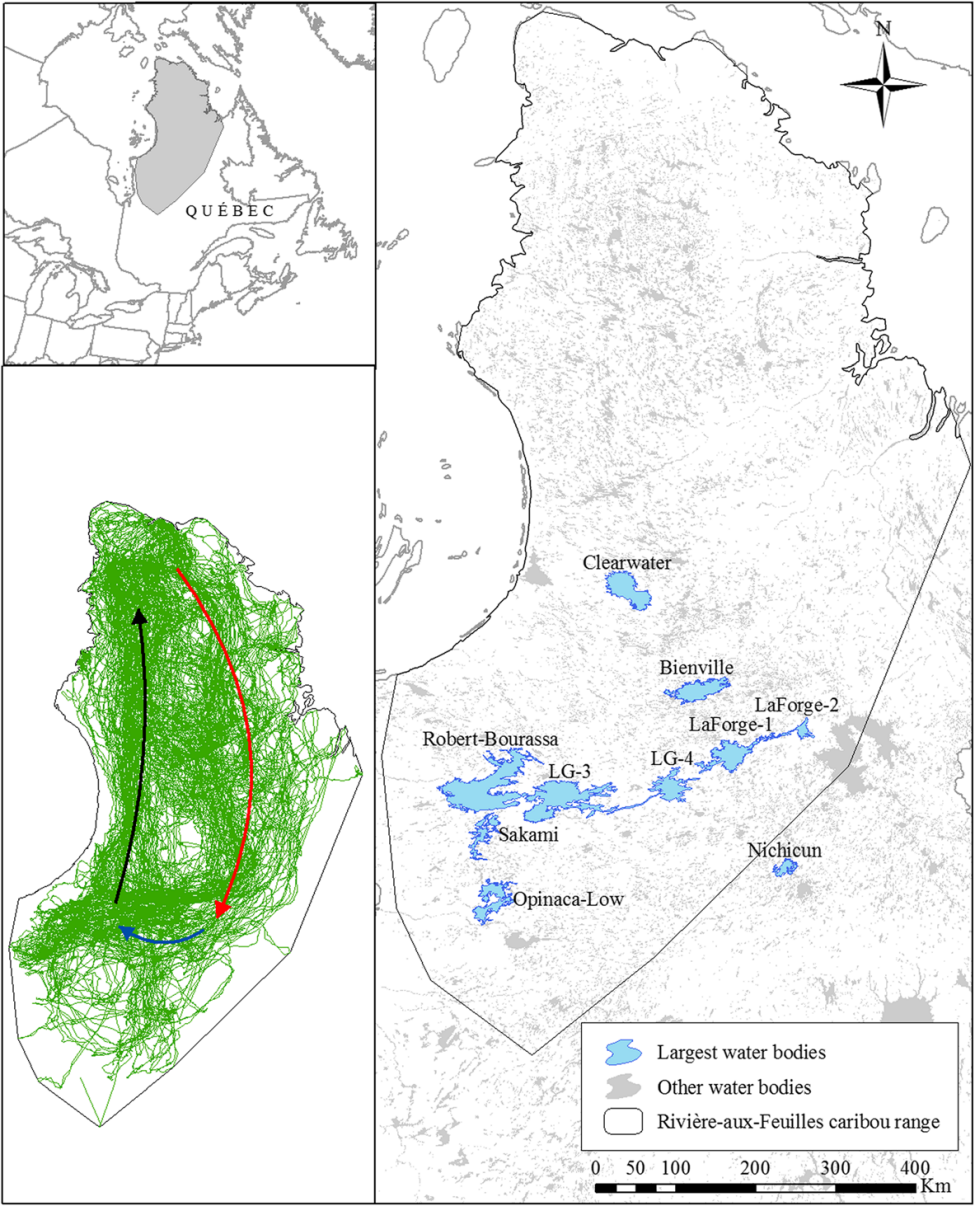
Study area in Northern Québec showing the range of the Rivière-aux-Feuilles caribou herd. We delineated the range using a 100 % minimum convex polygon encompassing caribou locations from 2007 to 2014. The largest water bodies used to study caribou responses to ice and water (as well as Lake Nichicun, see Additional file 3) appear in blue. All other water bodies in the province appear in light grey. Inserts show the location of our study area (*top-left*) and the steps performed by caribou during this study (*bottom-left*). Overlaid over observed steps (*green lines*) are the approximate trajectories of the fall migrations (*red arrow*), winter displacements (*blue arrow*), and spring migrations (*black arrow*) performed by caribou

The RAF caribou herd ranged over >630 000 km^2^ across Northern Québec, Canada (Fig. 1). Females gave birth in the Arctic tundra of the Ungava Peninsula (61°N, 74°W), generally between early and mid-June. In October–December, caribou undertook a long migration across the taiga and into the northern fringe of the boreal forest, where they fed on arboreal and terrestrial lichens (mostly *Cladonia*, *Cetraria*, and *Usnea*) found in open black spruce (*Picea mariana*) stands [33]. In April, they left their wintering ranges and initiated a migration back to the calving grounds. Across this entire journey, they coped with severe climatic conditions typical of arctic and subarctic regions [34]. In the southern part of the caribou range, a series of hydroelectric infrastructures were built during Hydro-Québec’s James Bay Project in the 1970s and 1980s. Many structures were built on La Grande River, along which several natural lakes were converted into some of the largest artificial reservoirs in the region (Fig. 1).

### Caribou data

From 2007 to 2014, we equipped 96 caribou (80 F, 16 M) from the RAF herd with GPS telemetry collars (Telonics, Mesa, AZ, USA; precision ≤20 m). The sample size was relatively modest at the onset of our study with seven individuals monitored in 2007 but increased consistently to reach 70 individuals monitored simultaneously in 2014, for a total of 181 individual-years (see Additional file 1 for more details). On average, we monitored 23 ± 8 SE (standard error) individuals each year, and individuals were monitored for 1.9 ± 0.1 SE years (up to six consecutive years). We captured caribou during winter using a net-gun fired from a helicopter. Capture procedures were approved by Animal Welfare Committees of the Ministère des Forêts, de la Faune et des Parcs du Québec and Université Laval (#2008-015 and 2011–039; certificates renewed each year). We programmed collars to record a location every 1, 2, 7, 11, or 13 h (every 4.2 ± <0.1 h, on average) depending on the period and collar model. For the purposes of a companion study [35], the frequencies of GPS locations were higher during migrations (from April to May for the spring migration and from October to December for the fall migration) and lower during summer and winter periods. We considered this variability in location frequency by accounting for time between successive locations in the analyses.

### Ice data

To assess fine-scale responses of caribou to the availability of ice on water bodies, we restricted our study to the vicinity of the largest lakes, rivers, and reservoirs in the study area. We used caribou monitoring data and digital hydrological maps to isolate large lakes that were susceptible to influence caribou behaviour. We selected only the largest water bodies (average of 1 475 km^2^, see Additional file 2) that were crossed by collared caribou during 2007–2014 because they could represent semipermeable barriers to caribou movements and induce locomotion costs. We also selected the largest lakes to ensure that crossing events were accurate, and not artefacts caused by the uncertainty of caribou trajectories between successive GPS locations.

### Thawing and freezing of water bodies

We estimated average thawing and freezing dates of water bodies between 2000 and 2014 using moderate-resolution imaging spectroradiometer (MODIS) maps generated by the National Snow & Ice Data Center [36]. MODIS maps are 500-m resolution grids based on a snow mapping algorithm that utilizes a normalized difference snow index averaged over 8 days starting in February 2000. We truncated MODIS data to the surface covered by the water bodies in a geographic information system (ArcGIS 10, ESRI Inc., Redlands, CA, USA), and for each 8-day period, we calculated the proportion of ice (ICE) to open water (WATER) using Eq. 1:

1 $$ \frac{no.\kern0.5em  of\kern0.5em  ICE\kern0.5em  cells\kern0.5em -\kern0.5em  no.\kern0.5em  of\kern0.5em  WATER\kern0.5em  cells}{no.\kern0.5em  of\kern0.5em  ICE\kern0.5em  cells\kern0.5em +\kern0.5em  no.\kern0.5em  of\kern0.5em  WATER\kern0.5em  cells} $$

**Fig. 2:**
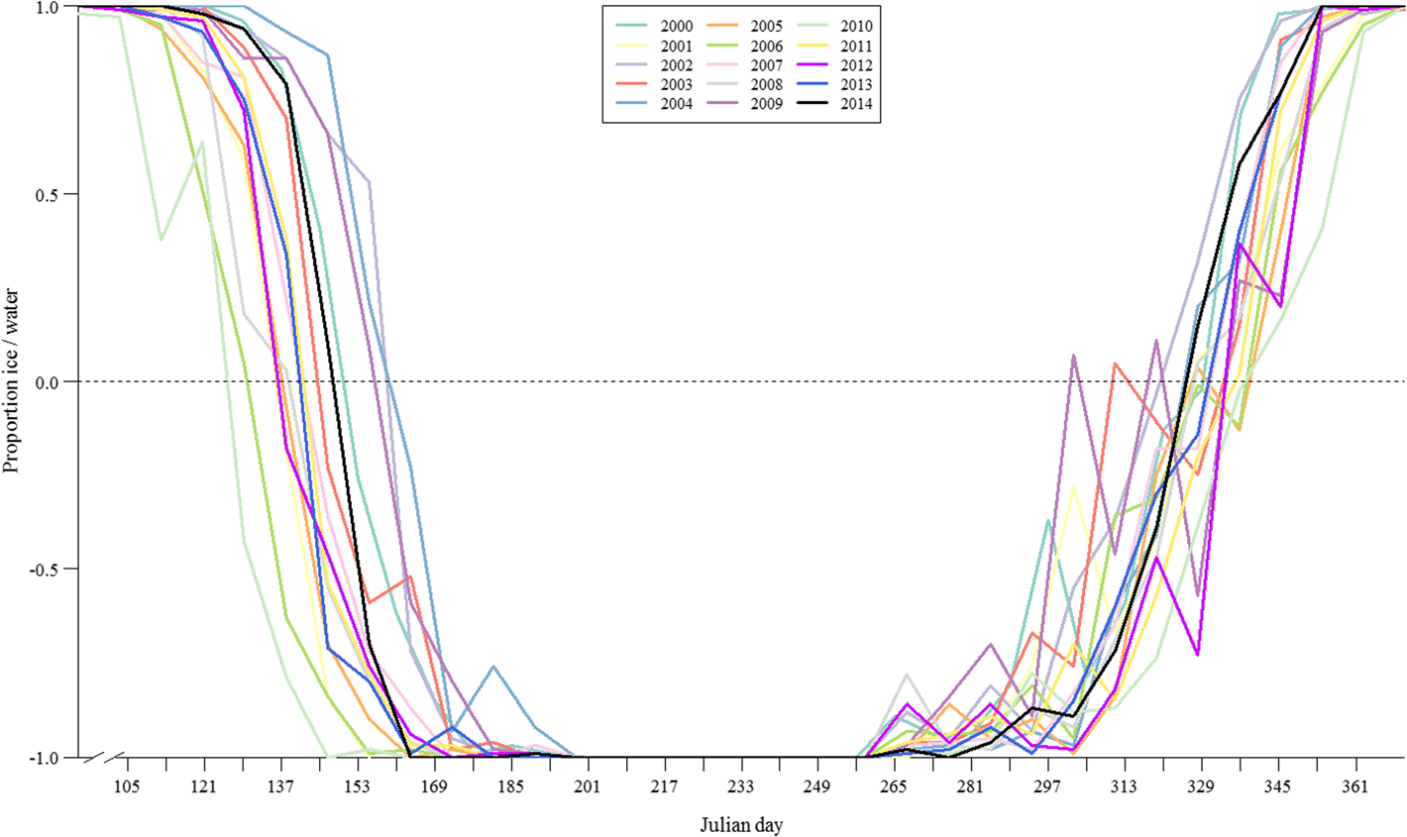
Proportion of ice and water on the largest water bodies used by migratory caribou. Ice and water coverages were estimated using 8-day averaged MODIS values from 2000 to 2014. Extreme values of -1.0 and 1.0 respectively represent open water and completely frozen water bodies

We only applied this equation to the cells of the MODIS raster grid that encompassed the retained water bodies. Other features (i.e., land, snow, and clouds) were assigned a null value. This equation generated values ranging between −1.0 (i.e., open water) and 1.0 (i.e., completely frozen). We plotted this index across years (Fig. 2), and used the Julian days at the x-intercepts as our reference points to study temporal trends between 2000 and 2014. These reference points represented dates when water bodies in our study area went from mostly frozen to mostly thawed (i.e., from a negative to a positive index value), and *vice versa*. We modeled lake thawing and freezing date trends during the 2000–2014 period using linear regressions in R 3.1.1 [37]. We also explored historical thawing and freezing trends in our region using data collected on Lake Nichicun (Northern Québec, Canada) from 1947 to 1985 (see Additional file 3). Finally, we assessed the relationship between annual breakup or freeze dates of the largest water bodies used by caribou (2000–2014) or of Lake Nichicun (1947–1985) and monthly values of broad-scale climatic oscillations, i.e., the North Atlantic Oscillation (NAO) and the Arctic Oscillation (AO; see Additional file 4).

### Caribou behaviour

We isolated GPS locations on the surface, as well as within 5-km buffer zones around the largest water bodies. We used these buffer zones to keep every location of individuals that reached the vicinity and either crossed or circumvented water bodies. We examined each sequence of successive locations (hereafter segments) to identify movements across surfaces (hereafter crossings) or around the periphery of lakes and rivers (hereafter detours). We were specifically interested in isolating the respective influence of ice, water, and land in facilitating or hindering caribou movements. We removed groups of successive clumped locations on land or islands (i.e., stopovers *sensu* [38]), which were probably representative of other activities like foraging or mating. We tested whether the relative frequency of water crossings increased with time using a linear regression. We also tested whether their relative frequency was explained by the availability of ice, by comparing years with early and late freezing dates using a Welch *t* test. Only freezing dates (not thawing dates) were used in these tests because water crossings only occurred during the fall.

### Caribou movement metrics

We discriminated movement segments (i.e., crossings and detours) using a minimum of three successive locations to allow the calculation of turning angles. We estimated turning angles (°) by measuring the angle between the first and second steps (i.e., steps are straight lines between successive locations) composing a segment. For segments longer than two steps, we calculated the mean turning angle across the whole segment. We also estimated the mean movement rate (m/h) across segments by averaging the Euclidian distance travelled between successive locations divided by the time the animal took to complete the step. Movement rates and turning angles were estimated using the *adehabitatLT* package in R [39]. We compared movement metrics of caribou on ice (*n* = 653 locations from 48 individuals), in water (*n* = 139 locations, 9 individuals), and during detours (*n* = 1 119 locations, 37 individuals) using linear mixed models with the Satterthwaite approximation for degrees of freedom in the *lmerTest* package [40]. We used all movements made by caribou at similar dates (i.e., between 16 September and 19 May) elsewhere in the study area as controls (*n* = 136 257 locations). We estimated models using individual × location frequency as a random effect. This was necessary to consider possible biases in the calculation of movement metrics across individuals having different location frequencies [41].

### Use and selection of water and ice by caribou

To assess the fine-scale availability of ice for caribou, we generated 1- and 2.5-km buffer zones around each location and evaluated the proportion of ice and water surrounding each location using the Geospatial Modelling Environment software [42]. These buffer zone sizes encompassed frequently reported zones of influence for *Rangifer* (e.g., [43, 44]). We then compared the composition of buffer zones using Welch *t* tests for crossings on ice, in water, and detours separately. Similar results were obtained using the 1- and 2.5-km buffer zones; thus, we only discuss results obtained using the 1-km buffer zone.

To evaluate the selection of ice and water by caribou, we performed a step selection function (SSF) using the *survival* package in R [45]. SSFs compare observed steps to random steps originating from the same location in a conditional regression framework [46]. We chose this method because the availability of ice and water needed to be measured in areas immediately available to individuals [47]. We paired observed steps to 10 random steps drawn from the frequency distributions of lengths and turning angles of each individual at each location frequency. Prior to estimating frequency distributions of step lengths, we removed the highest 5 % outlier distances to prevent generating overly long random steps [46]. The conditional regression evaluated the probability of observing a real step with varying proportions of ice and water along each step. Although many environmental variables other than water and ice availability drive fine-scale movements of ungulates [48], we refrained from complexifying models to specifically test our hypothesis that caribou avoided water and selected ice during step selection.

We assessed the influence of water body type (natural lake, river, or hydroelectric reservoir) in a separate model, by testing the interaction between water body type and the proportion of both ice and water along each step. We used a model composed of interactions with a 3-level water body type factor, but simpler dichotomies (i.e., natural lakes and rivers vs. reservoirs, lakes and reservoirs vs. rivers) gave similar results and are not shown. We compared the goodness of fit of models using Akaike’s information criterion corrected for small sample sizes (AIC_c_ [49]). To assess the fit of the most parsimonious SSF model, we ranked logit values predicted by the model for observed and control steps within each stratum (i.e., a stratum is one observed step and its 10 associated control steps) and performed a Spearman rank correlation between step ranks (i.e., 1–11, the latter rank having the highest logit) and the number of observed steps in each rank (see [48]). We performed *k*-fold cross validation using 80 % of strata as the training set and 20 % as the testing set, and report the average Spearman rank correlation ($\overline{r_S}$) resulting from 10 random draws of training and testing sets.

To measure possible inter-annual trends in caribou behaviour, we developed annual SSF models composed of the same covariates as the most parsimonious model for the whole study period. Year could not be included as a single covariate in a global SSF model, because conditional regressions compare strata at the scale of a step (i.e., always intra-annual). We also grouped years based on ice availability, isolating years with a short ice-availability period from years with a long ice-availability period, and used this dichotomy to evaluate separate SSF models. Year 2014 was not included in these analyses because caribou monitoring ended in summer 2014, before the freezing of water bodies.

### Consequences of future changes in ice phenology for caribou

We assessed the potential consequences of climate change for migratory caribou by simulating future changes in ice phenology in our study area. For each year with caribou data (2007 – 2014), we advanced the average breakup date of the largest water bodies by 1-day increments, up to a 30-day change. We performed the same simulations in fall by delaying the average freeze date by 1-day increments for 30 days. At each increment, we determined the proportion of ice crossings that would not have been possible if breakup had been advanced or freezing had been delayed by 1 day. We then compared these simulations to various lake-ice cover projections made across North America [19, 50–52]. All projections were based on the high-emission A2 scenario of the Intergovernmental Panel on Climate Change (the “business-as-usual” scenario [1]), but differed in their general circulation models. We calculated the mean predicted change in date for each projection (studies often reported a range of potential values or results from multiple climate models) and compared these values to our simulations. We used these projections to describe the potential availability of ice for caribou in our study area 25 – 50 years from now, i.e., in 2041–2070.

Using these projections and our monitoring data, we estimated the additional distance required to circumvent water bodies in the future by isolating all ice crossings that would have become impossible (due to the absence of ice) and replacing them with simulated detours around water bodies. To do so, we simply isolated the first and last locations of ice crossings and generated new paths around water bodies, using the shortest route possible. We then compared the distance travelled by caribou during our study to the predicted distance obtained under potential future climate conditions. We compared distances between segments using paired *t*-test and report the total difference in distance during the whole study. We did not generate detours from segments across rivers or leading to islands because finding an alternate route was often impossible.

## Results

### Thawing and freezing of water bodies

**Fig. 3:**
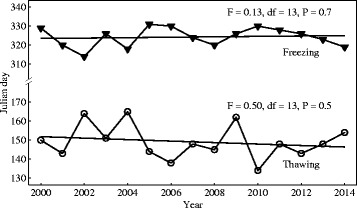
Trends in thawing and freezing dates for the largest water bodies used by migratory caribou. Thawing (*circles*) and freezing dates (*triangles*) were based on 8-day averaged MODIS values from 2000 to 2014. Values were truncated for the largest lakes, rivers, and reservoirs included in the range of the migratory Rivière-aux-Feuilles caribou herd (see list in Additional file 2). Regressions were: Thawing date = 930.4 ˗ 0.39(Year), and Freezing date = 94.9 + 0.11(Year)

Between 2000 and 2014, the largest water bodies used by caribou went from mostly frozen to mostly thawed as early as May 13^th^ (2010) and as late as June 13^th^ (2004), with an average thawing date of May 28^th^ (±8.8 days standard deviation, SD). Water bodies began freezing as early as November 9^th^ (2002) and as late as November 26^th^ (2005), with an average freezing date of November 19^th^ (±5.1 days SD). Windows of opportunity during which ice was available for caribou ranged from a maximum of 201 days in 2009 to a minimum of 168 days in 2010. There was no statistically significant trend in thawing and freezing dates during this 15-year period (thawing: F = 0.50, df = 13, *P* = 0.49; freezing: F = 0.13, df = 13, *P* = 0.72; Fig. 3). Similarly, no statistically significant trend was observed for Lake Nichicun during the 1947–1985 period (see Additional file 3). Breakup dates of the largest lakes used by migratory caribou and Lake Nichicun were related to the NAO in May, whereas their freeze dates were related to the NAO in October and the AO in September (see Additional file 4).

### Caribou behaviour

**Fig. 4:**
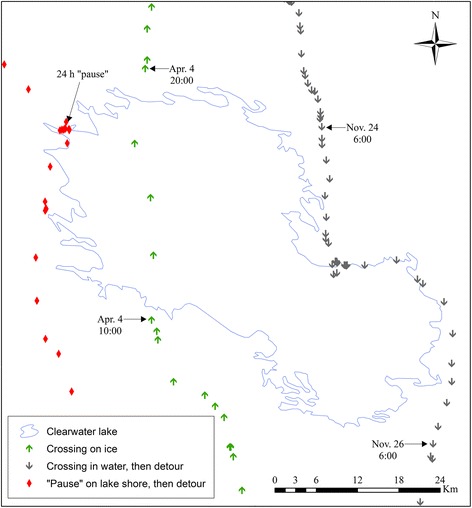
Examples of commonly observed caribou movements in the vicinity of large water bodies. Prolonged “pauses” on water body shores before or after crossings and detours are illustrated using red diamonds. Fast and directional movements on ice are illustrated using green arrows. Unsuccessful attempts at crossing open water lakes are illustrated using grey arrows. These movements were made by two migratory caribou from the Rivière-aux-Feuilles herd in the vicinity of Clearwater Lake between November 2010 and April 2014. Similar movements were observed on other water bodies at different dates by different individuals

Of the 181 individual-years monitored, 95 (52 individuals) used the vicinity (i.e., surfaces and 5-km buffer zones) of the largest water bodies in the study area. These individuals performed 179 ice crossings, 11 water crossings, and 129 detours. The longest crossing was recorded on reservoir Robert-Bourassa, when a caribou walked on ice for ~30 h over a >60 km distance. The caribou with the longest swimming distance swam across Lake Bienville over a >25 km distance. Based on MODIS maps, ice was completely unavailable during this crossing, which occurred on October 19^th^ ˗ 21^st^, 2013. All water crossings occurred during the fall migration (i.e., between 16 September and 29 November), and most of them occurred on the northernmost lakes (i.e., Clearwater and Bienville). The frequency of water crossings did not change with time (*F* = 1.98, df = 5, *P* = 0.22) or between years with early (2007–2008, and 2013) and late freezing (2009–2012; *t* = −0.95, df = 3.59, *P* = 0.40). During 5 of the 11 water crossings, caribou began crossing in water but eventually got out and went around instead (Fig. 4). Many individuals made long “pauses” before or after crossing or circumventing water bodies (Fig. 4). No collared caribou died on or in the vicinity of water bodies during our study.

### Movement metrics

**Fig. 5:**
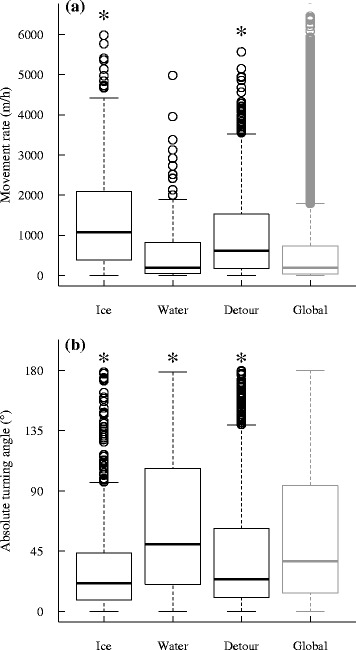
(**a**) Movement rates and (**b**) turning angles of migratory caribou across different substrates. Movements on and around water bodies are compared to movements elsewhere in the study area at similar periods, from 2007 to 2014. Statistical differences from the reference category (i.e., Global) are indicated using an asterisk

The movement rates of caribou (corrected for individual and location frequency) were significantly higher on ice (1625 ± 51 m/h SE; *t* = 31.95, df = 136 211, *P* < 0.01) and during detours (994 ± 31 m/h SE; *t* = 18.64, df = 136 163, *P* < 0.01) than elsewhere in the study area (535 ± 2 m/h SE; Fig. 5a). Movement rates across water were not different from controls (643 ± 82 m/h SE; *t* = 1.09, df = 136 146, *P* = 0.27). The turning angle (absolute value: range 0–180°) of steps was higher in open water (70.3 ± 4.7° SE; *t* = 2.14, df = 135 988, *P* = 0.03) and lower across ice (33.9 ± 1.5° SE; *t* = −11.23, df = 136 054, *P* < 0.01) and detours (43.3 ± 1.3° SE; *t* = −8.81, df = 136 006, *P* < 0.01) compared to all other steps at similar dates (57.6 ± 0.1° SE; Fig. 5b).

### Use and selection of water and ice by caribou

By examining the composition of 1-km buffer zones around caribou locations in the vicinity of water bodies, we determined that caribou tended to use ice when it was available: locations on ice were surrounded at 61.1 ± 1.7 % SE by ice and at 4.6 ± 0.7 % SE by water, on average (*t* = 30.8, df = 476, *P* < 0.01). When ice was not available, caribou used water, which occupied 62.4 ± 3.5 % SE of the area surrounding water-crossing locations, against 18.4 ± 3.2 % SE for ice (*t* = −9.3, df = 178, *P* < 0.01). Caribou made detours even when ice was available (i.e., the area surrounding detours was composed at 48.4 ± 2.3 % SE by ice and at 10.5 ± 1.6 % SE by water); however, proportions of ice and water surrounding detours differed significantly from movements across ice (*P* < 0.01), suggesting that caribou made more detours when ice was less available.

Caribou avoided open water and selected ice during their fine-scale movements in the vicinity of, and across water bodies (Table 1). The most parsimonious model performed especially well for a model that comprised only water and ice availabilities ($\overline{r_S}$ = 0.52). Interactions with water body type did not increase the goodness of fit, suggesting that caribou reacted similarly to large natural lakes, rivers, and reservoirs (Table 1). Results from the annual SSF models were similar to the global model performed on all years (Table 2). Significant effects (i.e., selection for ice and avoidance of water) were uniform across years and for years with short (2010–2012) and long ice-availability windows (2007–2009 and 2013). Avoidance of water was significant during years with a long ice-availability period, and selection for ice was significant during years with a short ice-availability period (Table 2).

**Table 1:** Results from global step selection function models. We present the parameter estimates (β) ± standard errors (SE) and *P* values of covariates included in step selection function (SSF) models explaining the crossings and detours of migratory caribou from the Rivière-aux-Feuilles herd in the vicinity of the largest lakes in Northern Québec, Canada. The least parameterized model, which is also the most parsimonious (lowest AIC_c_ value), appears in black, whereas the more complex model with interactions (and a higher AIC_c_ value) appears in grey. Number of parameters (*k*), log-likelihood (*LL*), and AIC_c_ values are shown for each model, and validation results ($\overline{r_S}$) appear for the most parsimonious model only

Model	Covariates	β ± SE	*P* value
Model 1, *k* = 2, *LL* = −3809.01, AIC_c_ = 7622.03, $\overline{r_S}$ = 0.52			
	Proportion of steps in water	−0.733 ± 0.363	**0.04**
	Proportion of steps on ice	0.475 ± 0.179	**0.01**
Model 2, *k* = 5, *LL* = −3808.28, AIC_c_ = 7628.57			
	Proportion of steps in water	−0.641 ± 0.469	0.17
	Proportion of steps on ice	0.581 ± 0.343	0.09
	Prop. water × Type:reservoir	−0.270 ± 0.786	0.73
	Prop. water × Type:river	0.663 ± 1.863	0.72
	Prop. ice × Type:reservoir	−0.087 ± 0.407	0.83
	Prop. ice × Type:river	−0.923 ± 0.872	0.29

Statistically significant effects (*P* ≤ 0.05) are shown in bold

**Table 2:**
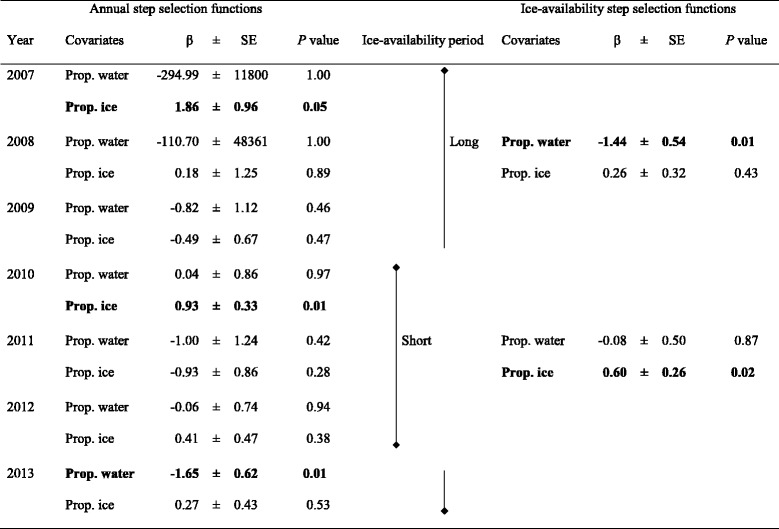
Results from annual step selection function models. We present the parameter estimates (β) ± standard errors (SE) and *P* values of covariates included in annual step selection function (SSF) models explaining the crossings and detours of migratory caribou from the Rivière-aux-Feuilles herd in the vicinity of the largest lakes in Northern Québec, Canada, from 2007 to 2013. Separate SSF models were also performed based on ice availability, isolating years with short (2010–2012) and long ice-availability periods (2007–2009, 2013)

### Consequences of future changes in ice phenology for caribou

**Fig. 6:**
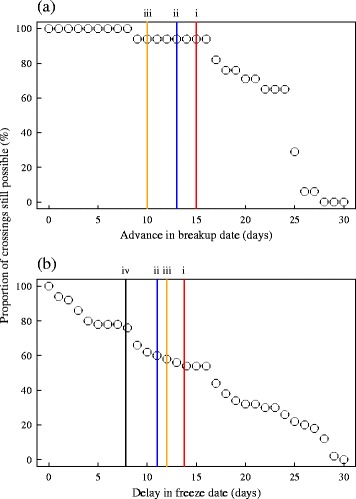
Relationship between (**a**) advance in breakup or (**b**) delay in freeze dates and the proportion of possible ice crossings. Ice crossings by migratory caribou were observed between 2007 and 2014, whereas changes in breakup or freeze dates (from 1 to 30 days) represent potential consequences of warming air temperatures in the Arctic during the next 25–50 years (2041–2070 horizon). Projections of future freeze and breakup dates are taken from (*i*) Brown and Duguay [19] in red, (*ii*) Dibike et al. [50] in blue, (*iii*) Shuter et al. [51] in orange, and (*iv*) Brammer et al. [52] in black

By simulating advances in the average thawing date of the largest water bodies in the caribou range, we found that only 6 % (1/17) of ice crossings performed during the spring migration would not have been possible if water bodies had melted 10–15 days earlier (Fig. 6a). When we delayed the average freezing date to projected values for the 2041–2070 horizon (delays of 7.8–13.8 days), the proportion of impossible ice crossings during the fall migration reached 24–46 % (12 to 23/50, Fig. 6b). Thus, future changes in ice phenology caused by a warming Arctic could result in the loss of as much as 36 % of ice crossings during the thawing and freezing periods by 2070.

Based on this result, we isolated all ice crossings performed by caribou during the first 15 days following freeze or preceding breakup and replaced them with detours (*n* = 17 instead of 24 because we excluded movements across rivers or leading to islands). We found that simulated detours were on average 2.6 times longer than their respective ice crossings (*t* = 4.7, df = 16, *P* < 0.01). Within this time frame, we found a ~28 % increase in the total distance travelled by caribou to circumvent water bodies. Specifically, the 27 detours and 23 ice crossings performed by caribou in the first 15 days preceding breakup or following freeze in 2007–2014 amounted to 888 km, whereas the 44 detours (including our 17 simulations) and 6 river/island crossings amounted to 1 133 km in the projected 2041–2070 conditions.

## Discussion

### Climate change and ice phenology

Declines in sea- [13, 14] and freshwater-ice [18, 19] have begun to affect the distribution and behaviour of arctic wildlife [15–17, 21]. Global surface temperatures have increased by approximately 0.12 °C (0.08 – 0.14 °C CI 90 %) per decade over the last >50 years (with Arctic warming going at least twice as fast [1]), and ice breakup and freeze dates on lakes and rivers in northern regions have changed correspondingly [18]. Temperatures are predicted to continue increasing in the future, and depending upon climate scenarios (see [1]), the average air temperature could rise 1.5 °C to 4.0 °C by the end of the 21^st^ century (with even more rapid changes in arctic/sub-arctic regions [11, 12]). These increases in the rate [1, 53] (see also [23, 54, 55] for comparable results on plant phenology) and magnitude [11, 12] of changes in arctic environments suggest that freshwater ice availability in Northern Québec will likely decrease in the foreseeable future. In fact, based on various projections across northern North America [19, 50–52], as much as 36 % of ice crossings performed by caribou during the early spring and fall migrations would not have been possible if icing phenology had been comparable to conditions anticipated for the 2041 – 2070 horizon.

Contrary to Magnuson et al. [18], we did not detect any significant icing trend on the largest water bodies used by caribou in Northern Québec (2000–2014) and Lake Nichicun (1947–1985). This study was based on a different methodology and used data collected over much broader spatial (i.e., Northern Hemisphere) and temporal (i.e., 19^th^ century onwards) scales. In comparison, our compilation of 15 years of MODIS data may have been insufficient to detect a significant trend. Broad scale predictive models usually outperform finer climatic models because of their capacity to capture a larger range of variability [1]. Other reports on local freshwater ice conditions have also demonstrated the difficulty of studying ice phenology at fine scales [50, 56]. Latifovic and Pouliot [56] found that, out of 36 lakes monitored across Canada from 1950 to 2004, 33 had earlier breakup and 24 had later freezing dates, but only 45 % and 42 % of these trends were statistically significant, respectively. The lack of additional long-term data on ice availability in our study area prevented us from performing more precise analyses.

### Caribou, water, and ice

As a consequence of a warming Arctic, caribou may encounter unfrozen lakes more frequently during their migrations. Ongoing research [M. Le Corre, C. Dussault, and S. D. Côté, unpublished observations] reveals that caribou in Northern Québec are initiating their spring and fall migrations earlier. This response may allow caribou to avoid a potential mismatch between the timing of migration and freshwater ice availability. Assuming caribou are unable to adjust the timing of their migrations further (e.g., see [23]), delays in ice phenology could force caribou to cross water bodies more frequently in open water or across ice too thin to bear their weight, causing increased risks of drowning [21]. Changes in ice phenology could also lead caribou to circumvent water bodies for several km, with possible negative consequences on their energy reserves. Considering that caribou rarely swam across unfrozen water bodies and frequently circumvented lakes for several km when ice was unavailable, the latter consequence seems likely.

Caribou in Northern Québec seemed to favour ice substrates during migrations and winter displacements. Their movements in water were slower and more sinuous than their movements on ice. The relatively long “pauses” caribou often made on lake shores could have allowed them to assess the quality (i.e., thickness) of the ice before crossing [21]. Caribou may also have used these “pauses” to feed and rest before or after crossing barren ice surfaces. Caribou made long excursions over frozen lakes, with the longest crossings exceeding 60 km. Reports of long journeys across ice by caribou and reindeer are not without precedent. Miller et al. ([57] and references therein) described observations of reindeer walking on ice for several hundreds of km, including a 380 km trip by one individual over the Barents Sea. These authors also observed reindeer remaining on ice for >25 km after land was easily accessible, suggesting that long trips across ice were not stressful for them. Based on the limited information about caribou swimming in the scientific literature, the longest water crossing we recorded was the longest ever reported (>25 km vs. 3–10 km [21, 58]). We cannot exclude the possibility, however, that the individual that performed this water crossing used islands smaller than the minimum resolution of digital maps (i.e., 500 m) as stopover sites.

Caribou swim at the water surface using quadrupedal paddling, which is amongst the least efficient types of swim in vertebrates [29]. Few comparative data exist for running and swimming energetics in fully-terrestrial mammals. Mink (*Neovison vison*) spend 2.7 times more energy swimming compared to running [59]. To cover a given distance, humans spend approximately four times more energy in water than on land, and go half as fast [60]. It is probable that the comparative costs of swimming vs. running for caribou are similar to those of humans, if not higher, due to the low propulsive surface area of their finer limbs. Thus, the avoidance of water and selection of ice by caribou could be largely explained by locomotion energetics.

## Conclusions

The movements of large herbivores are influenced by a suite of environmental and anthropogenic factors, including the type of substrate [47], topography [48], forage availability [38], and presence of human infrastructure [22]. More recently, the effects of broad-scale changes in climate on ungulate movements have also begun to be documented. For example, Stien et al. [7] demonstrated that Svalbard reindeer (*R.t. platyrhynchus*) increased their displacement distances following rain-on-snow events, which are predicted to occur more frequently during warm winters. In Northern Québec, we revealed a link between caribou movements and inter-annual variations in ice availability. Caribou avoided water and selected ice in years with long and short ice-availability periods, respectively, suggesting that caribou were able to respond to ice availability and, indirectly, prevailing climatic conditions. Ice availability could thus be one of many drivers explaining the distribution of caribou following climate change. Predicting future migration routes of caribou, however, remains challenging [61].

Other landscape features, notably anthropogenic disturbances, also have the potential to disrupt ungulate migrations [22]. Berger [26] observed that the ubiquity of human infrastructure like fences, highways, and oil drills forced pronghorn (*Antilocapra americana*) and mule deer (*Odocoileus hemionus*) of the Greater Yellowstone Ecosystem (Wyoming, USA) to migrate through relatively narrow bottlenecks surrounded by topographic and anthropogenic features. Although Northern Québec is much less disturbed than Wyoming [22, 26], the conservation of migratory caribou in Northern Québec is nonetheless believed to be constrained by increasing industrial development, human activities, and climate change [24]. Hydroelectric infrastructures, roads, mines, and buildings already occur throughout the area, and industrial developments, notably mineral exploration and exploitation, are expected to accelerate in the near future [62]. Along the large hydroelectric reservoirs of La Grande River, the land migration routes of caribou are located on isthmuses between water bodies. Provided that caribou will have fewer opportunities to cross and will circumvent reservoirs more often during migrations, interactions between migratory caribou and humans are bound to increase. Future studies will need to tackle the hard task of differentiating the relative contributions of all factors involved in the spatial shifts of arctic herbivores following global changes in climate and land use.

### Availability of supporting data

The dataset supporting the conclusions of this article is available in the Dryad Digital Repository (DOI:10.5061/dryad.4k275).

## Additional files

Additional file 1: GPS telemetry program on migratory caribou of the Rivière-aux-Feuilles herd between 2007 and 2014 in Northern Québec, Canada. (DOCX 14 kb) Additional file 2: Largest water bodies used by migratory caribou of the Rivière-aux-Feuilles herd between 2007 and 2014. (DOCX 15 kb) Additional file 3: Breakup and freeze dates of Lake Nichicun, Northern Québec, Canada, 1947–1985. (DOCX 40 kb) Additional file 4: Relationships between the annual breakup and freeze dates of the largest water bodies used by the Rivière-aux-Feuilles caribou and Lake Nichicun in Northern Québec, Canada, and monthly values of the North Atlantic and Arctic Oscillations. (DOCX 28 kb)
