# Supplementary material for: Caribou, water, and ice – fine-scale movements of a migratory arctic ungulate in the context of climate change
Source: Mov Ecol. 2016 Apr 20;4:14. doi: 10.1186/s40462-016-0079-4 (PMC4837602; doi:10.1186/s40462-016-0079-4)
Supplement: Additional file 1: — GPS telemetry program on migratory caribou of the Rivière-aux-Feuilles herd between 2007 and 2014 in Northern Québec, Canada. (DOCX 14 kb) [file 40462_2016_79_MOESM1_ESM.docx]

**Additional file 1.** GPS telemetry program on migratory caribou of the Rivière-aux-Feuilles herd between 2007 and 2014 in Northern Québec, Canada. The number of monitored individuals per year and the number of GPS locations per year × location frequency are shown. The location frequency of GPS collars varied with annual period and collar model.

|  |  | **No. of GPS locations**^a^ | | | | |
| --- | --- | --- | --- | --- | --- | --- |
|  |  | **Location frequency** | | | | |
| **Year** | **No. of individuals monitored** | **1 h** | **2 h** | **7 h** | **11 h** | **13 h** |
| 2007 | 7 | 0 | 0 | 3 364 | 253 | 0 |
| 2008 | 5 | 0 | 0 | 2 623 | 239 | 0 |
| 2009 | 17 | 6 063 | 7 | 883 | 294 | 0 |
| 2010 | 27 | 15 958 | 10 595 | 5 240 | 284 | 0 |
| 2011 | 23 | 20 908 | 10 819 | 7 430 | 195 | 0 |
| 2012 | 32 | 0 | 32 126 | 8 490 | 0 | 0 |
| 2013 | 60 | 0 | 34 833 | 11 315 | 0 | 3 219 |
| 2014 | 111 | 0 | 11 651 | 6 848 | 0 | 4 727 |

^a^ The number of GPS locations collected during the whole study period (between 16 September and 19 May, 2007 – 2014, see main text for details) was 136 257.
