# Supplementary material for: Caribou, water, and ice – fine-scale movements of a migratory arctic ungulate in the context of climate change
Source: Mov Ecol. 2016 Apr 20;4:14. doi: 10.1186/s40462-016-0079-4 (PMC4837602; doi:10.1186/s40462-016-0079-4)
Supplement: Additional file 2: — Largest water bodies used by migratory caribou of the Rivière-aux-Feuilles herd between 2007 and 2014. (DOCX 15 kb) [file 40462_2016_79_MOESM2_ESM.docx]

**Additional file 2.** Largest water bodies used by migratory caribou of the Rivière-aux-Feuilles herd between 2007 and 2014.

| **Water Body** | **Type** | **Area (km^2^)**^a^ | **Latitude** | **Longitude** |
| --- | --- | --- | --- | --- |
| Clearwater | Natural lake | 1 391 | 56°08’N | 74°18’W |
| Bienville | Natural lake | 1 187 | 55°04’N | 72°51’W |
| Laforge-2 | Reservoir | 281 | 54°40’N | 70°46’W |
| Laforge-1 | Reservoir | 1 277 | 54°21’N | 72°08’W |
| LG-4 | Reservoir | 956 | 53°59’N | 73°16’W |
| LG-3 | Reservoir | 2 788 | 53°50’N | 75°24’W |
| Robert-Bourassa | Reservoir | 3 749 | 53°45’N | 77°00’W |
| Sakami | Reservoir | 619 | 53°15’N | 76°45’W |
| Opinaca-Low | Reservoir | 1 021 | 52°40’N | 76°22’W |
| La Grande | River | NA | Laforge-2 to Robert-Bourassa | |

^a^ The area refers to the surface covered by water bodies on digital maps. This area may include islands, floodplains, shores, and land.
