# Supplementary material for: Caribou, water, and ice – fine-scale movements of a migratory arctic ungulate in the context of climate change
Source: Mov Ecol. 2016 Apr 20;4:14. doi: 10.1186/s40462-016-0079-4 (PMC4837602; doi:10.1186/s40462-016-0079-4)
Supplement: Additional file 3: — Breakup and freeze dates of Lake Nichicun, Northern Québec, Canada, 1947–1985. (DOCX 40 kb) [file 40462_2016_79_MOESM3_ESM.docx]

**Additional file 3. Breakup and freeze dates of Lake Nichicun, Northern Québec, Canada, 1947 – 1985.**

To explore historical icing trends in our study area, and because MODIS data were only available starting in 2000, we used the Canadian Lake Ice Database [1] available on the Polar Data Catalog [2] to determine average breakup and freeze dates of Lake Nichicun (311 km^2^) from 1947 to 1985. Lake Nichicun was not used by the Rivière-aux-Feuilles caribou herd between 2007 and 2014, but it was located just southeast of their range (see Figure 1 in main text). We considered breakup and freeze dates on this lake as representative of breakup and freeze dates for other large water bodies in the region during the same period.

**Methods**

Between 1947 and 1985, different observers stationed at Nitchequon station (53°11'N, 70°52'W) noted the breakup (n = 34 years) and freeze dates (n = 36 years) of Lake Nichicun. We modeled breakup and freeze dates using linear and piecewise (“broken stick”) regressions with the *SiZer* package [3] in R 3.1.1 [4]. To our knowledge, no data on ice extent were available in our study area between 1985 and 2000 or before 1947.

**Results**

Lake Nichicun broke up as early as May 28^th^ (1971) and as late as June 22^nd^ (1956), with an average breakup date of June 8^th^ (± 6.8 days SD). During the same period, it froze as early as October 12^th^ (1974) and as late as November 13^th^ (1955), with an average freeze date of October 30^th^ (± 7.8 days SD). There was no statistically significant trend during this 38-year period (*P* ≥ 0. 30; Figure AF3-1). Freeze dates decreased with time during the 1947-1975 period, but a statistical change was identified in 1976 after which freezing started to occur at later dates (Figure AF3-1).

**References**

1. Lenormand F, Duguay CR, Gauthier R. Development of a historical ice database for the study of climate change in Canada. Hydrological Processes. 2002;16(18):3707-22. doi:10.1002/hyp.1235.
2. Polar Data Catalogue. https://www.polardata.ca/. Accessed 2 November 2015.
3. Sonderegger D. SiZer: Significant zero crossings. R package version 0.1-4. 2012. url: CRAN.R-project.org/package=SiZer. Accessed 2 November 2015.
4. R Core Team. R: A language and environment for statistical computing. R Foundation for Statistical Computing. Vienna. url: https://www.r-project.org/. Accessed 2 November 2015.


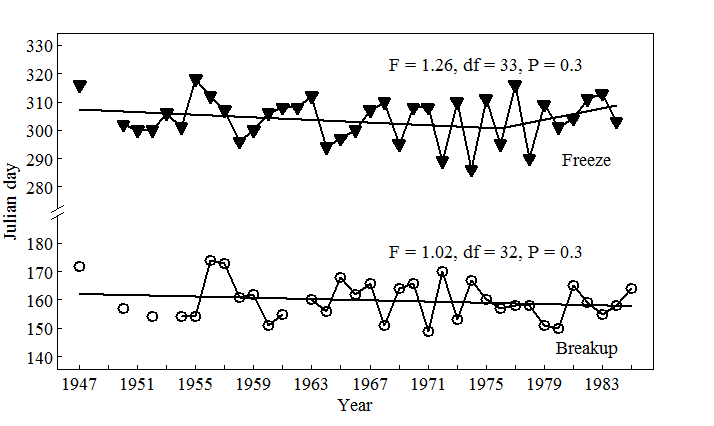
**Figure AF3-1.** Historical breakup (circles) and freeze dates (triangles) of Lake Nichicun, Northern Québec, Canada, 1947 – 1985. Regressions were: Breakup date = 383.0 ˗ 0.11(Year), and Freeze date = 760.2 ˗ 0.23(Year) + 1.23(Year ˗ 1976)^+^.
