# Supplementary material for: Caribou, water, and ice – fine-scale movements of a migratory arctic ungulate in the context of climate change
Source: Mov Ecol. 2016 Apr 20;4:14. doi: 10.1186/s40462-016-0079-4 (PMC4837602; doi:10.1186/s40462-016-0079-4)
Supplement: Additional file 4: — Relationships between the annual breakup and freeze dates of the largest water bodies used by the Rivière-aux-Feuilles caribou and Lake Nichicun in Northern Québec, Canada, and monthly values of the North Atlantic and Arctic Oscillations. (DOCX 28 kb) [file 40462_2016_79_MOESM4_ESM.docx]

**Additional File 4.** Relationships between the annual breakup and freeze dates of the largest water bodies used by the Rivière-aux-Feuilles caribou and Lake Nichicun in Northern Québec, Canada, and monthly values of the North Atlantic and Arctic Oscillations.

We evaluated the relationships between annual breakup and freeze dates of the largest lakes used by migratory caribou (2000 – 2014) of the Rivière-aux-Feuilles herd or Lake Nichicun (1947 – 1985) and monthly values of broad-scale climatic oscillations, i.e., the North Atlantic Oscillation (NAO) and the Arctic Oscillation (AO). Broad-scale climatic oscillations (e.g., NAO) have been shown to influence the growth, fecundity, and survival of Arctic herbivores, including caribou and reindeer (e.g., [1, 2]). These climatic oscillations integrate broad-scale weather conditions like temperature, precipitations, and the frequency of extreme weather events, which in turn may influence the availability and quality of vegetation, and, ultimately, trophic interactions among plants, herbivores, and large carnivores [3].

**Methods**

We obtained monthly NAO and AO values on the website of the National Oceanic and Atmospheric Administration [4]. We fitted linear regression models between the annual breakup date and NAO/AO values for April, May, and June, and between the annual freeze date and NAO/AO values for September, October, and November, respectively.

**Results**

Annual breakup and freeze dates of the largest water bodies in the caribou range (2000 – 2014) or of Lake Nichicun (1947 – 1985) correlated well with broad-scale climatic oscillations. Breakup dates were related to the NAO in May (F = 8.63, df = 46, *P* = 0.01), whereas freeze dates were related to the NAO in October (F = 11.46, df = 48, *P* < 0.01) and the AO in September (F = 9.37, df = 48, *P* < 0.01; see Figures AF4-1 to AF4-3). Correlations between broad-scale climatic oscillations and breakup/freeze dates of freshwater bodies in our study area were relatively similar to results obtained by Bonsal et al. [5] in the same region.

**References**

1. Post E, Stenseth NC. Climatic variability, plant phenology, and northern ungulates. Ecology. 1999;80(4):1322-39. doi: 10.1890/0012-9658(1999)080[1322:Cvppan]2.0.Co;2.
2. Joly K, Klein DR, Verbyla DL, Rupp TS, Chapin FS, III. Linkages between large-scale climate patterns and the dynamics of Arctic caribou populations. Ecography. 2011;34(2):345-52. doi:10.1111/j.1600-0587.2010.06377.x.
3. Post E, Forchhammer MC. Climate change reduces reproductive success of an Arctic herbivore through trophic mismatch. Philos T R Soc B. 2008;363(1501):2369-75. doi: 10.1098/rstb.2007.2207.
4. National Oceanic and Atmospheric Administration. http://www.noaa.gov/. Accessed 2 November 2015.
5. Bonsal BR, Prowse TD, Duguay CR, Lacroix MP. Impacts of large-scale teleconnections on freshwater-ice break/freeze-up dates over Canada. J Hydrol. 2006;330(1-2):340-53. doi:10.1016/j.jhydrol.2006.03.022.

**Figure AF4-1.** Relationship between the annual breakup date (in Julian day) and the North Atlantic Oscillation (NAO) in May for the largest lakes used by migratory caribou of the Rivière-aux-Feuilles herd in Northern Québec, Canada (2000 – 2014) and Lake Nichicun (1947 – 1985).

**Figure AF4-2.** Relationship between the annual freeze date (in Julian day) and the North Atlantic Oscillation (NAO) in October for the largest lakes used by migratory caribou of the Rivière-aux-Feuilles herd in Northern Québec, Canada (2000 – 2014) and Lake Nichicun (1947 – 1985).

**Figure AF4-3.** Relationship between the annual freeze date (in Julian day) and the Arctic Oscillation (AO) in September for the largest lakes used by migratory caribou of the Rivière-aux-Feuilles herd in Northern Québec, Canada (2000 – 2014) and Lake Nichicun (1947 – 1985).
